# Rapid screening of critically ill patients for low plasma vitamin C concentrations using a point-of-care oxidation–reduction potential measurement

**DOI:** 10.1186/s40635-021-00403-w

**Published:** 2021-08-09

**Authors:** Sander Rozemeijer, Bob Smit, Paul W. G. Elbers, Armand R. J. Girbes, Heleen M. Oudemans-van Straaten, Angelique M. E. de Man

**Affiliations:** 1 Department of Intensive Care Medicine, Amsterdam UMC, Location VUmc, De Boelelaan 1117, 1081 HV Amsterdam, The Netherlands; 2 Research VUmc Intensive Care (REVIVE), 1081 HV Amsterdam, The Netherlands; 3 Amsterdam Medical Data Science (AMDS), 1081 HV Amsterdam, The Netherlands; 4 Amsterdam Cardiovascular Science (ACS), 1081 HV Amsterdam, The Netherlands; 5 Amsterdam Infection and Immunity (AII), 1081 HV Amsterdam, The Netherlands; 6 LabWest, Haga Teaching Hospital, Els Borst-Eilersplein 275, 2545 AA The Hague, The Netherlands

**Keywords:** Vitamin C deficiency, Ascorbic acid, Plasma vitamin C concentration, Reactive oxygen species, Oxidative stress, Oxidation–reduction potential, Point-of-care device, Surrogate marker, Screening tool, Model

## Abstract

**Background:**

Hypovitaminosis C and vitamin C deficiency are common in critically ill patients and associated with organ dysfunction. Low vitamin C status often goes unnoticed because determination is challenging. The static oxidation reduction potential (sORP) reflects the amount of oxidative stress in the blood and is a potential suitable surrogate marker for vitamin C. sORP can be measured rapidly using the RedoxSYS system, a point-of-care device. This study aims to validate a model that estimates plasma vitamin C concentration and to determine the diagnostic accuracy of sORP to discriminate between decreased and higher plasma vitamin C concentrations.

**Methods:**

Plasma vitamin C concentrations and sORP were measured in a mixed intensive care (IC) population. Our model estimating vitamin C from sORP was validated by assessing its accuracy in two datasets. Receiver operating characteristic (ROC) curves with areas under the curve (AUC) were constructed to show the diagnostic accuracy of sORP to identify and rule out hypovitaminosis C and vitamin C deficiency. Different cut-off values are provided.

**Results:**

Plasma vitamin C concentration and sORP were measured in 117 samples in dataset 1 and 43 samples in dataset 2. Bias and precision (SD) were 1.3 ± 10.0 µmol/L and 3.9 ± 10.1 µmol/L in dataset 1 and 2, respectively. In patients with low plasma vitamin C concentrations, bias and precision were − 2.6 ± 5.1 µmol/L and − 1.1 ± 5.4 µmol in dataset 1 (*n* = 40) and 2 (*n* = 20), respectively. Optimal sORP cut-off values to differentiate hypovitaminosis C and vitamin C deficiency from higher plasma concentrations were found at 114.6 mV (AUC 0.91) and 124.7 mV (AUC 0.93), respectively.

**Conclusion:**

sORP accurately estimates low plasma vitamin C concentrations and can be used to screen for hypovitaminosis C and vitamin C deficiency in critically ill patients. A validated model and multiple sORP cut-off values are presented for subgroup analysis in clinical trials or usage in clinical practice.

**Supplementary Information:**

The online version contains supplementary material available at 10.1186/s40635-021-00403-w.

## Introduction

Vitamin C plasma concentrations are often decreased in critically ill patients [1]. Low concentrations are associated with endothelial dysfunction, organ failure and mortality [2–4]. Intravenous administration of vitamin C may be beneficial [5], but results vary among clinical studies [6–18]. Vitamin C administration may especially be beneficial at lower plasma concentrations. Hypovitaminosis C (< 23 µmol/L) and vitamin C deficiency (< 11 µmol/L) are commonly used cut-off values to describe low vitamin C status. However, these cut-off values come from old literature and are arbitrary chosen to determine scurvy [19]. Reasonably, a more gradual impact of decreasing plasma vitamin C concentrations on clinical outcome is expected. Unfortunately, low vitamin C status often goes unnoticed, because determination of the plasma vitamin C concentrations is challenging. Blood samples need to be stabilized quickly and the laboratory analysis is laborious, expensive and not available for routine care. Therefore, a novel way to screen patients rapidly for low plasma vitamin C concentrations is relevant for both clinicians who consider vitamin C administration and researchers who want to perform a stratified trial analysis. A potential suitable marker for this purpose is the static oxidation reduction potential (sORP), which reflects the amount of oxidative stress in the blood.

In critically ill patients huge amounts of reactive oxygen species (ROS) and reactive nitrogen species (RNS) are generated [20, 21]. Vitamin C is our primary circulating antioxidant [22] and is metabolically consumed if oxidative stress is high. The total amount of oxidative stress will therefore have a significant impact on the total amount of vitamin C, vice versa*.*

The RedoxSYS system (Aytu Bioscience, Englewood, CO, USA), a point-of-care device, is able to measure the static oxidation reduction potential (sORP) within 20 min in a sample volume of 30 µL [23–25]. It measures the balance between the total amount of oxidants and reductants in the plasma. sORP appeared to be useful for the estimation of plasma vitamin C concentrations in critically ill patients and healthy volunteers, as shown in our recently published retrospective study using thawed plasma samples [23]. A strong negative relation was found between sORP and plasma vitamin C concentration.

The primary aim of this study is to prospectively validate our previous findings in fresh samples and to validate a model that can estimate vitamin C status. The secondary aim of the study is to determine the diagnostic accuracy of sORP to discriminate between low and higher plasma vitamin C concentrations. sORP cut-off values will be provided for the conventionally used cut-offs of both hypovitaminosis C, and vitamin C deficiency. The repeatability of the sORP measurement and the impact of freezing and storage on absolute sORP results were investigated first.

## Methods

### Study design

This study was performed in a mixed medical/surgical adult intensive care unit (ICU) of the Amsterdam University Medical Centers, Location VUmc, Amsterdam, the Netherlands. The study was approved by the local Ethics Board (registration NL66863.029.18, decision 2018.502). Written informed consent was obtained from all participants or their legal representatives prior to inclusion.

Eligible for inclusion were patients admitted to the ICU with systemic inflammatory response syndrome (SIRS), sepsis [26], trauma or after cardiac arrest. These patient categories are ‘at risk’ for low plasma vitamin C concentrations [1, 3, 23, 27].

### Vitamin C measurements

Heparinized blood samples were obtained on the day of admission (day 1) and on day 3 if the patient was still in the ICU. After centrifugation (10 min, 1800G), the supernatant was stabilized with 5.6% meta-phosphoric acid (1:5) and frozen at − 80 °C until vitamin C measurement by high-performance liquid chromatography–ultraviolet (HPLC–UV). Total vitamin C was measured (the sum of ascorbic acid (AA) and dehydroascorbic acid (DHA), an oxidation product of AA). No clinical information was available to the assessors of the vitamin C measurement.

### sORP measurements

Samples were collected with heparin. After centrifugation (10 min, 1800G), sORP was directly measured in the plasma samples. sORP was measured without knowledge about patients’ plasma vitamin C concentration. The total time from obtaining blood until a sORP result is less than 20 min. A detailed description of the sORP measurement by the RedoxSYS System (Aytu Bioscience, Englewood, CO, USA) has previously been outlined [23–25]. To assess the effect of a freeze–thaw cycle and storage on the variability of the measurement, sORP was measured again in 30 plasma samples (duplicates) that had been stored at − 80 °C for 6 months. Prior to the sORP measurements, a calibration verification test was performed and each new lot of sensors was verified with an external control solution (Zobell’s solution). sORP was also directly measured in 28 samples with acidified heparinized plasma, serum and EDTA–plasma. These samples were all obtained at the same time as 28 heparinized blood samples. Results are shown in Additional file 2: Table S1 to show the differences in absolute sORP values. These results are beyond the scope of this paper.

### Defining hypovitaminosis C and vitamin C deficiency

Hypovitaminosis C and vitamin C deficiency are defined as a plasma vitamin C concentration lower than 23 and 11 µmol/L, respectively, as currently used in daily clinical practice [1]. Plasma vitamin C concentrations in healthy volunteers become saturated at about 70–80 μmol/L with an intake of around 0.2 g/day [28].

### Statistics

All data were analyzed using IBM SPSS Statistics version 26. Normality was tested using skewness results, histograms and the Shapiro–Wilk test. Normally distributed variables are reported as mean ± standard deviation (SD) and not normally distributed variables as median [25th to 75th] percentile. A *p*-value of < 0.05 was considered to be statistically significant.

### Impact of storage

First, the repeatability of the sORP measurement was assessed by calculating the coefficient of variation (CV, %) of 10 random triplicate fresh measurements. Second, the variability in sORP results due to freezing and storage was assessed by calculating the mean difference in sORP between the 30 fresh and thawed duplicate samples (sORP of thawed samples minus the sORP of fresh samples). Bland–Altman plots, one-sample *T*-tests and linear regression models were used to visualize and calculate the mean bias and proportional bias. Subsequently, a model validation was carried out.

### Model validation

The underlying aim of this study is to *estimate* plasma vitamin C concentration from the sORP, as measured directly at the bedside (point of care). In our previous work [23], a logarithmic function was computed to estimate sORP from plasma vitamin C concentration. In this study, we recalculated the best fit of the relationship between plasma vitamin C concentration as dependent variable and sORP as independent variable. This exponential function was then used for model validation: ${\text{plasma }} {\text{vitamin }} {\text{concentration}}= 785.19 {e}^{-0.030 {\text{sORP}}}$. The model was validated by determining its accuracy in two separate datasets as described below.

The accuracy of this model, in terms of bias and precision, was assessed in accordance with the ISO 5725 standard [29]. Bias is the systematic error between the estimated plasma vitamin C concentration, based on our model, and the measured plasma vitamin C concentration by HPLC (gold standard). Bias was calculated as the mean difference between both measurements (measurement of plasma vitamin C by HPLC (gold standard) minus estimated plasma vitamin C by sORP measurement). Bland–Altman plots and linear regression models were used to visualize and calculate the proportional bias. In case of proportional bias, a subgroup analysis on patients with low plasma vitamin C concentrations (< 23 µmol/L, as measured with HPLC) was carried out. The precision of our model is the random (non-systematic) error of individual measurements and is quantified as the standard deviation (SD) of the bias and limits of agreement (1.96 SD). Thus, a high accuracy is the result of the combination between a low bias and high precision. The accuracy of the model was visualized in a Bland–Altman plot.

The accuracy was assessed in dataset 1 (training set), in which our exponential model was constructed, containing 117 thawed plasma samples from healthy volunteers and critically ill patients [23] and in dataset 2 (validation set), containing freshly obtained plasma samples from critically ill patients. In dataset 2, very high plasma vitamin C concentrations (> 150 µmol/L) were excluded as our model estimates plasma vitamin C concentration up to approximately 150 µmol/L [23]. In dataset 1, healthy volunteers and critically ill patients were both included, explaining the relatively high plasma concentrations. Dataset 2 only consists of critically ill patients whose plasma concentrations are within a clinically relevant range. Three patients received vitamin C therapy and were excluded as their plasma vitamin C concentrations were > 150 µmol/L.

### Diagnostic accuracy of sORP

A receiver operating characteristic (ROC) curve and area under the curve (AUC) were constructed to show the ability of sORP to correctly identify or rule out the *actual* measured hypovitaminosis C and vitamin C deficiency by HPLC in both datasets separately and combined. The sensitivity, specificity, positive and negative predictive values (PPV and NPV) were calculated for different cut-off points. The cut-off points with a 100% sensitivity and with a 100% specificity were identified, as well as the optimal cut-off points using Youden’s J Statistic. The 41 healthy volunteers from dataset 1 were not included in these analyses, as they were not ‘at risk’ for low plasma vitamin C concentrations.

## Results

Twenty-five different patients were enrolled for dataset 2. Baseline characteristics of the patients included in both datasets are shown in Table 1.

**Table 1:** Baseline characteristics

Patients	Dataset 2	Dataset 1^a^
	Total (*n* = 25)	Total (n = 42)
Age (years)	70 (56–76)	61 (48–76)
Sex, male (%)	15 (60)	27 (64.3)
BMI (kg/m^2^)	26.3 (23.5–30.6)	25.3 (22.4–27.6)
SIRS/sepsis/trauma (n) Cardiac arrest (n)	14 (56%) 11 (44%)	26 (61.9%) 16 (38.1%)
Lactate day 1 (mmol/L)	3.7 (2.4–4.9)	2.4 (1.7–4.8)
SOFA day 1^b^	8 ± 3	7 ± 3
APACHE II	28 (22–30)	28 (22–31)^c^
APACHE III	104 (71–123)	102 (73–122)
Plasma vitamin C concentration (µmol/L) day 1	28.5 (14.8–44.3)	25.3 (16.0–36.0)
sORP (mV) day 1	114.2 (98.7–131.9)	114.2 (102.2–126.9)^d^
Plasma vitamin C concentration (µmol/L) day 3	23.0 (12.1–53.6)^e^	18.7 (13.7–28.8)^f^
sORP (mV) day 3	121.3 (102.3–135.8)^e^	119.4 (107.8–135.5)^g^

*APACHE* Acute Physiology and Chronic Health Evaluation; *BMI* body mass index; *SIRS* systemic inflammatory response syndrome; *sORP* static oxidation–reduction potential; *SOFA* Sequential Organ Failure Assessment

Data are presented as mean ± standard deviation or as median with (interquartile range)

^a^Previously published data [23]. The authors gave consent for republishing the baseline characteristics

^b^SOFA-scores are calculated without the central nervous system score due to its unreliability when patients receive sedatives. SOFA-scores were calculated according to the NICE criteria [30]

^c^Measured in 40 patients

^d^Measured in 41 patients

^e^Measured in 21 patients

^f^Measured in 36 patients

^g^Measured in 35 patients

### Plasma vitamin C concentration and sORP measurements

In dataset 2, plasma vitamin C concentration was measured in 25 patients at day 1 and 21 patients at day 3. 3 out of 46 samples were excluded as their plasma vitamin C concentrations were > 150 µmol/L due to vitamin C therapy. In the remaining 43 samples, sORP was measured directly.

### Repeatability of the RedoxSYS system in fresh plasma

In a random subset of 10 samples of dataset 2, sORP was measured three times to assess the repeatability of the RedoxSYS System. The coefficient of variation was 4.6% (sORP range from 74.0 mV to 148.6 mV).

### The impact of storage at − 80 °C

**Fig. 1:**
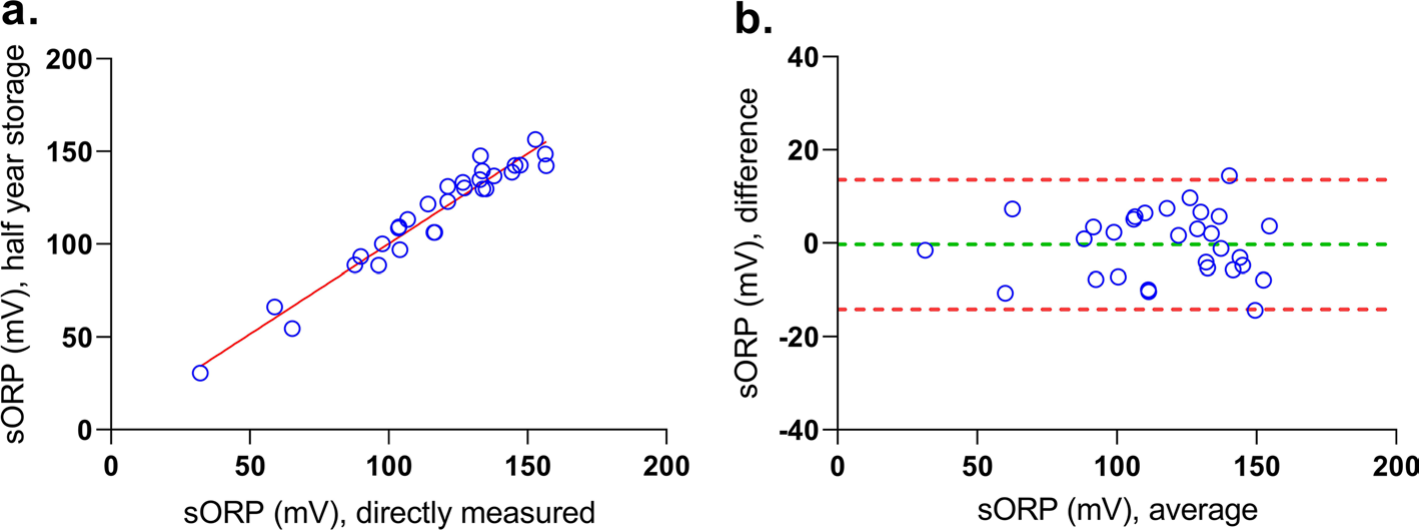
Correlation (left) and Bland–Altman (right) plot. **a** The *X*-axis represents the sORP results of direct measurements and the *Y*-axis represents the stored samples (*R*^2^ linear = 0.94). **b** The *X*-axis represents the average sORP of the direct and half year measurements. The *Y*-axis represents the difference of the two measurements. The mean bias (green line) and its confidence limits (limits of agreement) (red lines) are shown

In 30 different samples of dataset 2, sORP was measured directly and after 6 months of storage at − 80 °C. When comparing the sORP results after 6 months with the direct sORP measurements, an average difference of − 0.3 ± 7.1 mV was found (Fig. 1). There was no statistically significant difference between the two measurements (*p* = 0.8). Linear regression and visual inspection by Bland–Altman analysis showed that there was no indication of a proportional bias (*p* = 1.0).

### Model accuracy

**Fig. 2:**
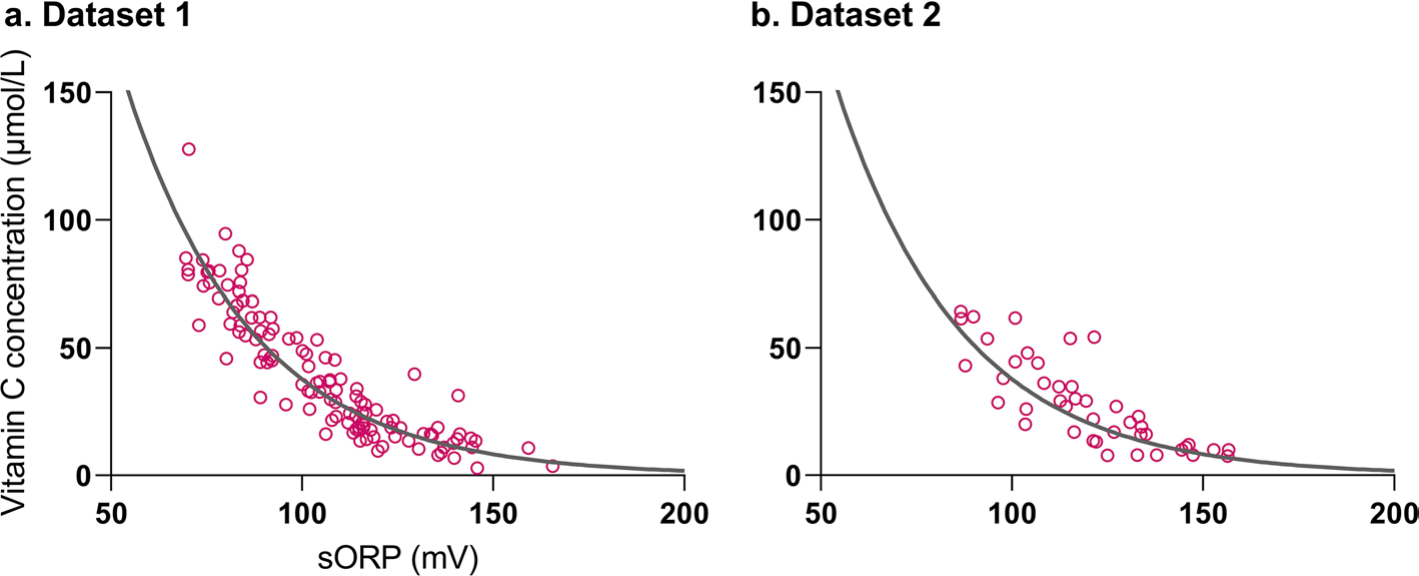
Scatter plot with exponential model in thawed plasma samples (n = 117), dataset 1 (**a**), and in fresh plasma samples (*n* = 43), dataset 2 (**b**). Equation: ${\text{plasma }} {\text{vitamin }} {\text{concentration}}=785.19 {e}^{-0.030 sORP}$

In dataset 2, 43 measurements were included. The exponential model has been plotted in both datasets (Fig. 2). A sORP of 116.3 mV and 140.5 mV correspond with an *estimated* plasma vitamin C concentration of 23 and 11 µmol/L, respectively.

**Fig. 3:**
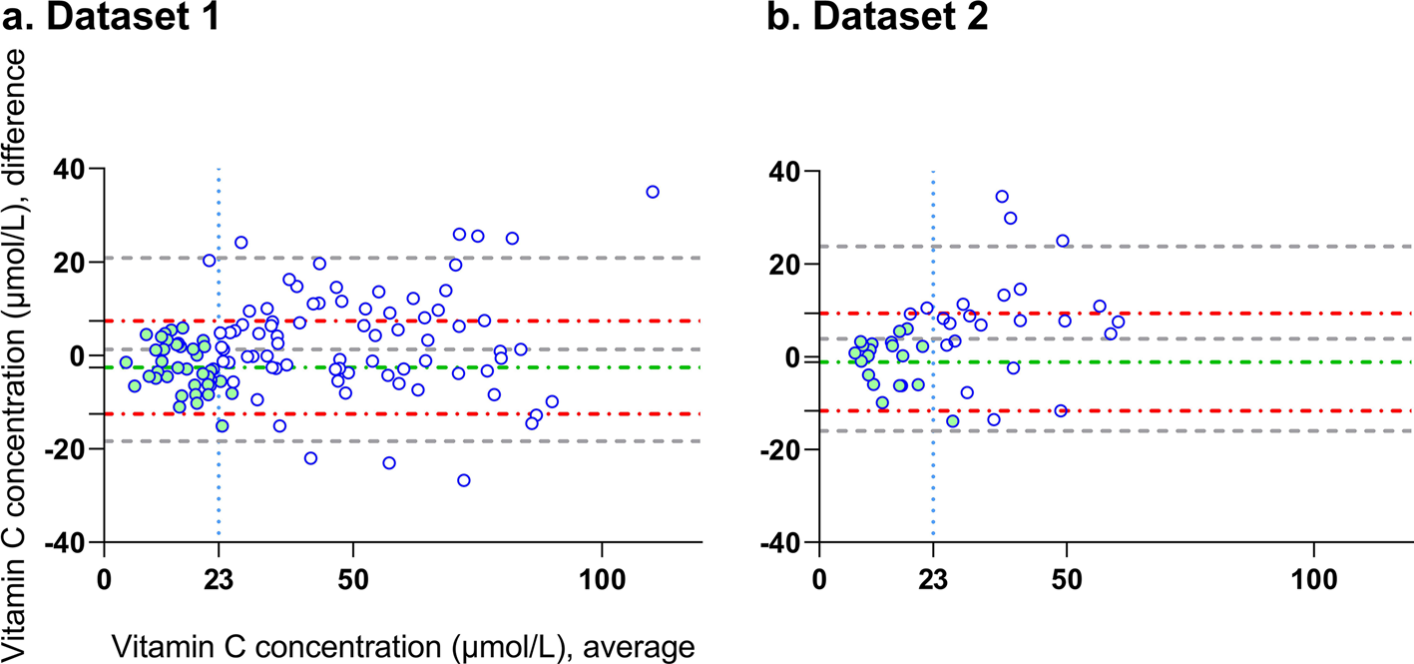
Bland–Altman plots of dataset 1 (**a**) and dataset 2 (**b**). The X-axes represent the average of the estimated plasma vitamin C concentration by our model and the measured plasma vitamin C concentration with HPLC (gold standard). The *Y*-axes represent the difference between the two measurements. The mean bias and its confidence limits (limits of agreement, LoA) are shown for the whole group (gray striped lines) and subgroup (< 23 µmol/L, green filled dots) analysis (mean bias = green dotted striped line and LoA = red dotted striped lines)

The bias (mean difference) and precision (SD) were 1.3 ± 10.0 µmol/L (95% CI − 0.6 to 3.1 µmol/L) and 3.9 ± 10.1 µmol/L (95% CI 0.7 to 7.0 µmol/L) in dataset 1 and 2, respectively. In both datasets a proportional bias was found (dataset 1: *p* = 0.03, and dataset 2: *p* = 0.02); see Fig. 3. A subgroup analysis on patients with low plasma vitamin C concentrations (< 23 µmol/L) showed a bias and precision of − 2.6 ± 5.1 µmol/L (95% CI − 4.2 to − 1.0 µmol/L) and − 1.1 ± 5.4 µmol/L (95% CI − 3.6 to 1.4 µmol/L) in dataset 1 (*n* = 40) and 2 (*n* = 20), respectively. The accuracy of our model in the whole groups and subgroups is shown in Fig. 3.

### Diagnostic accuracy of sORP: cut-off value determination and interpretation

**Fig. 4:**
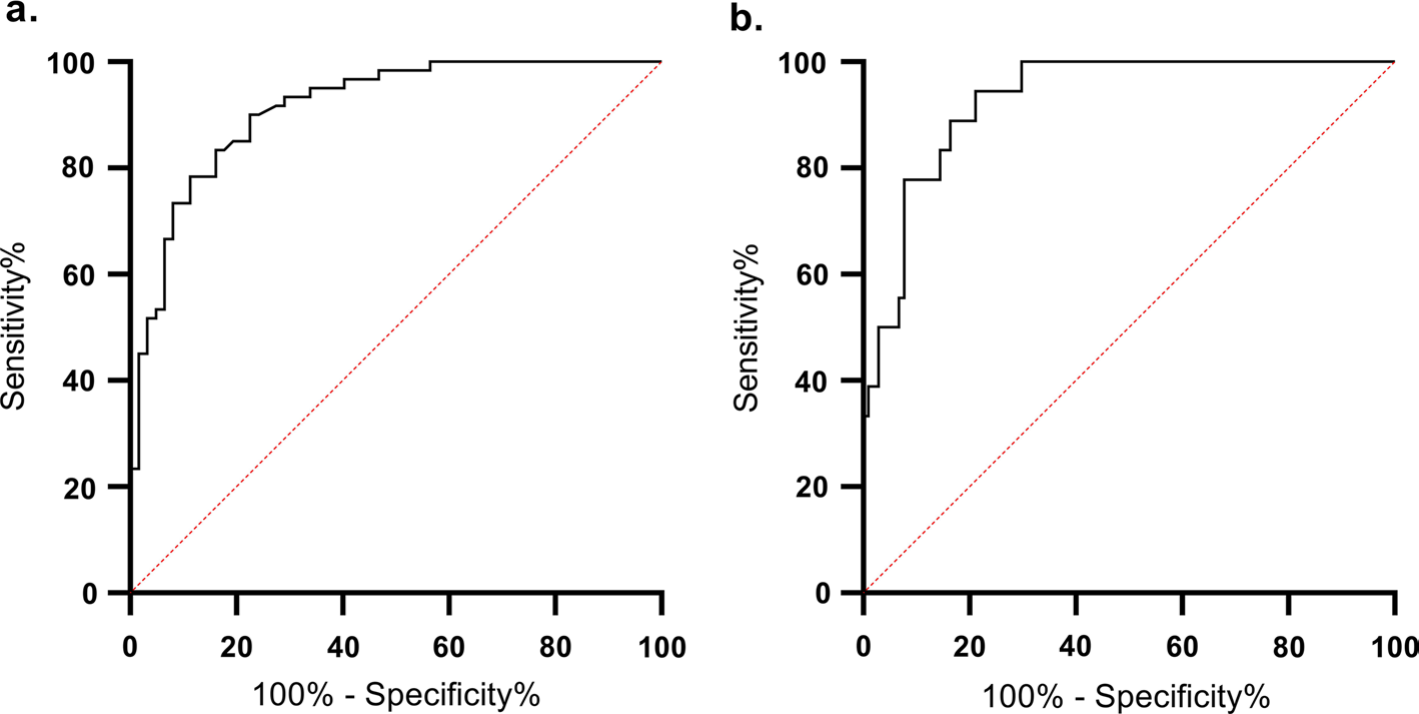
ROC-curves for hypovitaminosis C (**a**) and vitamin C deficiency (**b**) in the combined datasets

The ROC-curves and different sORP cut-off values for hypovitaminosis C and vitamin C deficiency are shown in Fig. 4 and Table 2 for both datasets combined. All patients with a sORP lower than 103.0 mV and 119.6 mV had plasma vitamin C concentrations > 23 and > 11 µmol/L, respectively. In addition, all patients with a sORP higher than 141.1 mV and 146.9 mV had hypovitaminosis C and vitamin C deficiency, respectively. The optimal cut-off point for hypovitaminosis C and vitamin C deficiency was found at a sORP value of 114.6 mV and 124.7 mV, respectively.

**Table 2:** sORP cut-off values for hypovitaminosis C and vitamin C deficiency

	Cut-off value, mV	Sensitivity, % (95% CI)	Specificity, % (95% CI)	PPV, % (95% CI)	NPV, % (95% CI)	AUC (95% CI)	p-value
Both fresh and thawed samples (datasets combined)							
Hypovitaminosis C (prevalence: 60/122)	**103.0**	100 (94–100)	44 (31–57)	63 (53–73)	100 (87–100)	0.91 (0.86–0.96)	< 0.001
	**114.6**^**a**^	90 (80–96)	77 (65–87)	79 (68–88)	89 (77–96)		
	**141.1**	23 (13–36)	100 (94–100)	100 (77–100)	57 (48–67)		
Vitamin C deficiency (prevalence: 18/122)	**119.6**	100 (82–100)	70 (60–79)	37 (23–52)	100 (95–100)	0.93 (0.88–0.98)	< 0.001
	**124.7**^**a**^	94 (73–100)	79 (70–86)	44 (28–60)	99 (94–100)		
	**146.9**	33 (13–59)	100 (97–100)	100 (54–100)	90 (83–95)		

*AUC* area under the curve, *CI* confidence interval, *NPV* negative predictive value, *PPV* positive predictive value

^a^Optimal cut-off value, as chosen using Youden’s J Statistic

The ROC-curves and the sORP cut-off values for the separate datasets are presented in Additional file 1: Fig. S1 and Additional file 3: Table S2.

## Discussion

This present study demonstrates that the static oxidation–reduction potential (sORP), as rapidly measured with a point-of-care device, can be used to accurately estimate low plasma vitamin C concentrations in both thawed and fresh plasma samples. Furthermore, this study illustrates that sORP can be used to screen for hypovitaminosis C and vitamin C deficiency in critically ill patients, and presents sORP cut-off values for both.

This is the first study that determined the applicability of the Redox SYS system to estimate patients’ plasma vitamin C concentration in freshly obtained blood samples. In both datasets, the results show that our model estimated patients’ plasma vitamin C concentration with an acceptable bias. However, the estimation was not equally precise for different plasma concentrations. The precision was higher at lower plasma vitamin C concentration. Based on the bias and precision in both datasets, we consider our model to be accurate for estimating decreased plasma vitamin C concentrations. To provide health care professionals with additional guidance while estimating the vitamin C status of their patients, multiple sORP cut-off values are presented. Our findings suggest that sORP has a very good capability to differentiate hypovitaminosis C and vitamin C deficiency from higher plasma concentrations in critically ill patients. It is important to emphasize the distinction between our validated model and the sORP cut-off values. The model *estimates* a plasma vitamin C concentration based on a sORP result, whereas the provided test characteristics in Table 2 are based on the *actual* vitamin C status of the tested study population (diagnostic accuracy of sORP). When a sORP of 114 mV is measured in a new patient, the *estimated* vitamin C is 25.7 µmol/L. However, the *actual* vitamin C status may be slightly different. Table 2 shows that at this sORP result around 10% of the tested study population had a plasma vitamin C concentration < 23 µmol/L (NPV around 89%).

Previous clinical studies showed that sORP is related to outcome and can be used to monitor the amount of oxidative stress in cardiac surgical patients [31] and patients with heart failure, sepsis and trauma [20, 25, 32–37]. In a previous study, we demonstrated that sORP can be used as a rapid and cheap surrogate marker to monitor changing plasma vitamin C concentrations due to disease and vitamin C supplementation in critically ill patients [23]. In vitro evidence already described a change (decrease) of sORP after adding ascorbic acid [24, 25, 38, 39].

In our retrospective cohort study, sORP was measured in thawed plasma samples, which were stored for 5 years. Therefore, any impact of a freeze–thaw cycle, storage and measurement error on sORP results could not be excluded. In this present study, no difference in sORP results was found after 6 months storage at − 80 °C, compared to direct measurements. Furthermore, our model had a comparable accuracy in both thawed and fresh samples. Both these findings show that sORP measurements are stable during frozen storage of plasma samples. This is in line with previous literature [24].

### Strengths

First, our study provides a validated model that estimates plasma vitamin C concentration which can be used to monitor patients’ vitamin C status. Second, multiple sORP cut-off values, in fresh and thawed samples, are provided. Besides an optimal cut-off point based on Youden’s index, a sORP cut-off value with a sensitivity of 100% and one with a specificity of 100% are presented. In this way, the presence of low plasma vitamin C concentrations can be made unlikely on the one hand (NPV of 100%) and the presence of hypovitaminosis C and vitamin C deficiency more likely on the other hand (PPV of 100%).

### Limitations

First, our model that estimates plasma vitamin C concentration up to approximately 150 µmol/L appeared to be more accurate for lower plasma vitamin C concentrations. With an average precision of ±5 µmol/L for estimations < 23 µmol/L, it will still be difficult to precisely determine very low plasma concentrations. However, a very precise estimation in this very low range is not needed in clinical practice, as the primary interest is to differentiate low from high concentrations with satisfactory validity. In addition, the lower precision (SD of 10 µmol/L) in the high vitamin C range is clinically irrelevant. Second, it is important to note that our model is designed to predict total vitamin C (the sum of ascorbic acid (AA) and dehydroascorbic acid (DHA), an oxidation product of AA). Some laboratories do not measure total AA but only AA. As a consequence, their reported plasma vitamin C concentrations are slightly lower. In our data (*n* = 46), the amount of AA was, on average, 90.8% of the total AA. Third, due to the different sample sizes of the datasets, the diagnostic accuracy of sORP has to be interpreted with a relative uncertainty, dependent on the sample size. Therefore, 95% confidence intervals are provided to give more insight in the precision whereby the sensitivity, specificity, PPV and NPV are estimated. Due to the lower sample size of dataset 2, the imprecision is larger compared to dataset 1 (Additional file 3: Table S2).

### Clinical implications

The sORP, as measured by the RedoxSYS system, can now be considered in daily clinical practice for estimation and monitoring of patients’ plasma vitamin C concentration, especially in the low range (< 23 µmol/L). Furthermore, the multiple sORP cut-off values enable researchers to perform subgroup analyses in order to identify subsets of patients who might or might not benefit from vitamin C therapy or give support to clinicians who already consider vitamin C therapy. In this regard, the prevalence of the vitamin C status is relevant. In our tested study population, the prevalence of hypovitaminosis C is 49% and vitamin C deficiency 15%. In case of low disease prevalence, the positive predictive value (PPV) of a test will be low, even when the test has a high specificity. As a consequence, sORP is not very suitable to identify vitamin C deficiency with a prevalence of 15%. However, it is suitable for ruling out vitamin C deficiency at this low prevalence because of the high sensitivity and, consequently, the high negative predictive value (NPV). When the prevalence of a disease becomes higher, the PPV increases and the test becomes more suitable to identify the disease, e.g., hypovitaminosis C in our study. Measuring sORP only at patients ‘at risk’ for low plasma vitamin C concentrations (e.g., septic shock) will increase the prevalence, and thus affect the way sORP results can be used.

Due to the instability of vitamin C, rapid oxidation of vitamin C is inevitable. Therefore, it essential to process blood samples quickly in order to get reliable sORP results. In this present study, we were able to measure sORP and store duplicates within 15 min after drawing blood. Both the basic sample processing and the short waiting time until a sORP result are in contrast to the laborious stabilization of the samples and costly HPLC method, as currently used for plasma vitamin C determination. Finally, all our measurements were performed in heparin plasma samples. When using other collecting tubes, the sORP results might differ from heparin (see Additional file 2: Table S1).

## Conclusion

Our present study shows that the RedoxSYS analyzer can now be used as a rapid screening tool that gives health care professionals an indication of the plasma vitamin C concentration of their patients within 20 min, by measuring the static oxidation reduction potential (sORP). A validated model that accurately estimates low plasma vitamin C concentration has been developed for daily usage. In addition, sORP cut-off values of 114.6 mV and 124.7 mV provide a very good capability to differentiate hypovitaminosis C and vitamin C deficiency from higher plasma vitamin C concentrations, respectively.

## Supplementary Information

**Additional file 1.** **Fig. S1.** ROC-curves for hypovitaminosis C (**a**.) and vitamin C deficiency (**b**.). Dataset 1 (blue line), dataset 2 (green line) and both datasets combined (black line) are shown.

**Additional file 2: Table S1.** sORP measured in different collecting tubes obtained at the same time

**Additional file 3: Table S2.** sORP cut-off values for hypovitaminosis C and vitamin C deficiency in the different datasets

## Data Availability

The datasets will be available from the corresponding author on reasonable request.

## Abbreviations

AA: Ascorbic acid
APACHE: Acute Physiology and Chronic Health Evaluation
AUC: Area under the curve
BMI: Body mass index
CI: Confidence interval
CV: Coefficient of variation
DHA: Dehydroascorbic acid
EDTA: Ethylenediaminetetraacetic acid
HPLC–UV: High-performance liquid chromatography–ultraviolet
ICU: Intensive care unit
MPA: Metaphosphoric acid
NPV: Negative predictive value
PPV: Positive predictive value
RNS: Reactive nitrogen species
ROC: Receiver operating characteristic
ROS: Reactive oxygen species
SD: Standard deviation
SIRS: Systemic inflammatory response syndrome
sORP: Static oxidation–reduction potential
SOFA: Sequential Organ Failure Assessment
