# Supplementary material for: Rapid screening of critically ill patients for low plasma vitamin C concentrations using a point-of-care oxidation–reduction potential measurement
Source: Intensive Care Med Exp. 2021 Aug 9;9:40. doi: 10.1186/s40635-021-00403-w (PMC8349944; doi:10.1186/s40635-021-00403-w)
Supplement: Supplementary file 2 — Additional file 2: Table S1. sORP measured in different collecting tubes obtained at the same time [file 40635_2021_403_MOESM2_ESM.docx]

| **Table S1. sORP measured in different collecting tubes obtained at the same time** | | | | |
| --- | --- | --- | --- | --- |
|  | Heparin plasma | Heparin plasma acidified with 5.6% MPA | Serum | EDTA-plasma |
| Directly measured (n=28) | 121.3 (103.6-137.2) | 433.7 (416.2-446.8) | 122.4 (101.1-137.5) | 141.4 (119.0-153.3) |
| Abbreviations: EDTA: Ethylenediaminetetraacetic acid; MPA: Metaphosphoric acid.  **Data are presented as median with (interquartile range).** | | | | |

**Supplementary Table 1**
