# Supplementary material for: Rapid screening of critically ill patients for low plasma vitamin C concentrations using a point-of-care oxidation–reduction potential measurement
Source: Intensive Care Med Exp. 2021 Aug 9;9:40. doi: 10.1186/s40635-021-00403-w (PMC8349944; doi:10.1186/s40635-021-00403-w)
Supplement: Supplementary file 3 — Additional file 3: Table S2. sORP cut-off values for hypovitaminosis C and vitamin C deficiency in the different datasets [file 40635_2021_403_MOESM3_ESM.docx]

| **Table S2. sORP cut-off values for hypovitaminosis C and vitamin C deficiency in the different datasets** | | | | | | | |
| --- | --- | --- | --- | --- | --- | --- | --- |
|  | Cut-off value, mV | Sensitivity, % (95% CI) | Specificity, % (95% CI) | PPV, % (95% CI) | NPV, %  (95% CI) | AUC  (95% CI) | p-value |
| Thawed samples (dataset 1) | | | | | | | |
| Hypovitaminosis C (prevalence: 40/76) | **105.5** | 100 (91-100) | 53 (36-70) | 70 (57-82) | 100 (82-100) | 0.907 (0.840-0.975) | <0.001 |
|  | **114.6^a^** | 88 (73-96) | 81 (64-92) | 83 (69-93) | 85 (69-95) |  |  |
|  | **141.1** | 18 (7-33) | 100 (90-100) | 100 (59-100) | 52 (40-64) |  |  |
| Vitamin C deficiency (prevalence: 10/76) | **119.6** | 100 (69-100) | 73 (60-83) | 36 (19-56) | 100 (93-100) | 0.921 (0.854-0.988) | <0.001 |
|  | **130.0^a^** | 90 (56-100) | 85 (74-93) | 47 (25-71) | 98 (91-100) |  |  |
|  | **145.6** | 30 (7-65) | 100 (95-100) | 100 (29-100) | 90 (81-96) |  |  |
| Fresh samples (dataset 2) | | | | | | | |
| Hypovitaminosis C  (prevalence: 20/46) | **102.2** | 100 (83-100) | 46 (27-67) | 59 (41-75) | 100 (74-100) | 0.937 (0.867-1.000) | <0.001 |
|  | **120.4^a^** | 90 (68-99) | 89 (70-98) | 86 (64-97) | 92 (74-99) |  |  |
|  | **133.4** | 55 (32-77) | 100 (87-100) | 100 (72-100) | 74 (57-88) |  |  |
| Vitamin C deficiency  (prevalence: 8/46) | **123.5^a^** | 100 (63-100) | 76 (60-89) | 47 (23-72) | 100 (88-100) | 0.937 (0.863-1.000) | <0.001 |
|  | **146.9** | 50 (16-84) | 100 (91-100) | 100 (40-100) | 91 (77-97) |  |  |
| Both fresh and thawed samples (datasets combined) | | | | | | | |
| Hypovitaminosis C  (prevalence: 60/122) | **103.0** | 100 (94-100) | 44 (31-57) | 63 (53-73) | 100 (87-100) | 0.913 (0.864-0.962) | <0.001 |
|  | **114.6^a^** | 90 (80-96) | 77 (65-87) | 79 (68-88) | 89 (77-96) |  |  |
|  | **141.1** | 23 (13-36) | 100 (94-100) | 100 (77-100) | 57 (48-67) |  |  |
| Vitamin C deficiency  (prevalence: 18/122) | **119.6** | 100 (82-100) | 70 (60-79) | 37 (23-52) | 100 (95-100) | 0.930 (0.881-0.979) | <0.001 |
|  | **124.7^a^** | 94 (73-100) | 79 (70-86) | 44 (28-60) | 99 (94-100) |  |  |
|  | **146.9** | 33 (13-59) | 100 (97-100) | 100 (54-100) | 90 (83-95) |  |  |
| Abbreviations: AUC: Area under the curve; CI: Confidence interval; NPV: Negative predictive value; PPV: Positive predictive value.  ^a^ Optimal cut-off value, as chosen using Youden’s J Statistic | | | | | | | |

**Supplementary Table 2**
